# Anodal high-definition transcranial direct current stimulation reduces heart rate and modulates heart-rate variability in healthy young people: A randomized cross-controlled trial

**DOI:** 10.3389/fcvm.2022.1070157

**Published:** 2022-12-02

**Authors:** Zhongke Gu, Wenxiang Chen, Qian Lu, Jiansong Dai, Shugang Hu, Kai Xu, Yao Geng, Ye Zhu, Boqing Xu, Wenjun Dai, Ying Shen

**Affiliations:** ^1^Department of Sport and Health Sciences, Nanjing Sport Institute, Nanjing, China; ^2^Department of Rehabilitation, Children's Hospital of Nanjing Medical University, Nanjing, China; ^3^Department of Rehabilitation Medicine, The Affiliated Jiangsu Shengze Hospital of Nanjing Medical University, Suzhou, China; ^4^Department of Rehabilitation, The Affiliated Jiangning Hospital With Nanjing Medical University, Nanjing, China; ^5^Rehabilitation Medicine Center, The First Affiliated Hospital of Nanjing Medical University, Nanjing, China

**Keywords:** high-definition transcranial current stimulation, cardiac rhythm, heart rate, heart-rate variability, dorsolateral pre-frontal cortex

## Abstract

**Objective:**

To investigate whether anodal high-definition transcranial current stimulation (HD-tDCS) over the left dorsolateral pre-frontal cortex (DLPFC) could modulate the heart rate (HR) and heart-rate variability (HRV) in healthy young people.

**Methods:**

Forty healthy young people were enrolled in this randomized crossover trial. The participants were randomized to receive anodal HD-tDCS (n = 20) or sham HD-tDCS (*n* = 20) over the left DLPFC with a washout period of 1 week. Electrocardiogram (ECG) data were continuously recorded 20 min before the stimulation, during the session (20 min), and 20 min after the session. HR and the time- and frequency-domain indices of the HRV were measured to investigate the activity of the sympathetic and parasympathetic nervous systems.

**Results:**

Anodal HD-tDCS over the left DLPFC induced a significant decrease in HR and a significant increase in the average of normal-to-normal intervals (AVG NN), low-frequency (LF) power, total power (TP), and LF/high-frequency (HF) ratio in comparison with the sham stimulation and the baseline. However, sham HD-tDCS over the left DLPFC had no significant effect on HR or HRV.

**Conclusions:**

Anodal HD-tDCS over the left DLPFC could reduce HR and modulate the HRV in healthy young people. HD-tDCS may show some potential for acutely modulating cardiovascular function.

## Introduction

The concept of the brain-heart pathway has been proposed by several recent studies (1, 2). The identification of dynamic and ongoing neural communication pathways between the heart and brain has elucidated the influence of the brain on cardiovascular responses in different conditions (3). Central commands originating in the cortical or subcortical areas and descending to the medulla oblongata through the spinal cord and extrinsic cardiac ganglia supervise the autonomic nervous outflow to modulate cardiac responses (4, 5). Regulation of the heart is known to involve the efferent pathways in the autonomic nervous system (5, 6). It has been demonstrated that brain-heart communication might regulate the level of cortisol in hypothalamic-pituitary-adrenal (HPA) axis activity via an indirect pathway to affect the cardiovascular system (7, 8). As outcome measures for evaluating the cardiac response, heart rate (HR) and heart-rate variability (HRV) have emerged as key parameters that reflect autonomic nervous system activity and cardiovascular health status (9, 10).

Functional neuroimaging studies have demonstrated the involvement of the pre-frontal cortex (PFC), anterior cingulate cortex, insula, and thalamus in brain-heart communication (11, 12). The PFC plays a regulatory role in cardiovascular voluntary activity as well as in the control of the autonomic nervous system (ANS) and HPA axis (13, 14). In their study on the adjustment of cardiac autonomic activity by the cortical and subcortical regions, Gianaros observed that the regional cerebral blood flow (rCBF) obtained by positron emission tomography was significantly reduced in the medial pre-frontal, insular, and anterior cingulate cortices with a reduction in HRV, which was positively correlated (12). Sakaki and colleagues concluded that in younger and older adults, the larger the HRV, the stronger the functional connection between the medial pre-frontal regions and the amygdala (15). Thus, the PFC was considered as a core region regulating HRV, which was verified by a meta-analysis of the brain regions associated with HRV (16).

Considering the involvement of some of these cortical regions in cardiovascular regulation, non-invasive brain stimulation (NIBS) may be useful to study their effects as a practical tool to investigate cardiac responses (9). Transcranial direct current stimulation (tDCS), a NIBS technique, could induce a long-term potentiation (LTP)-like facilitation or long-term depression (LTD)-like suppression (17, 18). This technique utilizes a constant, low-intensity direct current (1-2 mA) to modulate the excitability of the brain cortex (19). Depending on the polarity of electrodes placed on the scalp, tDCS shows bidirectional regulation in the excitability of neurons in neural networks (20). Anodal tDCS can depolarize the membrane potential, activate Na^+^ and Ca^2+^ voltage-gated channels, improve neuronal excitability, and enhance cortical activity (21). While conventional tDCS has an insufficient spatial resolution, high-definition tDCS (HD-tDCS) performed using small ring electrodes instead of two large pad electrodes can stimulate target specific brain areas accurately and produce notable after-effects (22, 23).

To date, limited research has focused on the effects of NIBS on the cardiac response and the efficacy of this technique, and the results of these studies were frequently not conclusive (24). In a meta-analysis, Maximilian demonstrated that tDCS alters the HR and HRV with small-to-medium effect sizes (9). Studies have shown that anodal tDCS (1.5 mA) over the left DLPFC of healthy volunteers gives rise to an increase in HF values and a decrease in cortisol levels (25). Meanwhile, Raimundo et al. found that the autonomic functions of healthy individuals who received anodal tDCS (1 mA) over the left primary motor cortex did not demonstrate any significant changes in blood pressure, HR or ventilatory rate (26). Given the region-specific differences in HR and HRV measures, these paradoxical results on the effects of tDCS may be explained. Therefore, in this study, we performed anodal HD-tDCS over the left DLPFC in healthy young individuals with the aim of investigating the influence of HD-tDCS on cardiac responses. We hypothesized that anodal HD-tDCS over the left DLPFC in healthy young individuals would reduce HR and modulate HRV.

## Materials and methods

### Participants

A total of 40 volunteers (14 males, 26 females) participated in the experiment. The inclusion criteria were as follows: (1) Zung Self-Rating Anxiety Scale (SAS) score < 50; (2) Zung Self-Rating Depression Scale (SDS) score < 53; (3) Athens Insomnia Scale (AIS) score < 4; (4) provision of voluntary written informed consent. Exclusion criteria were as follows: (1) a history of insomnia; (2) a history of neurological or psychiatric conditions; (3) a history of severe head injury; (4) use of any psychoactive medication; (5) a history of cardiac disease; (6) allergy to direct current.

### Sample size

The sample size was determined using the Gpower program. Based on published literature (27), power, alpha, and effect size were set at 95%, 0.05, and 1.45, respectively, calculating that 18 subjects were required for a single group. To allow for an anticipated dropout rate of 10%, there were a total of 40 subjects for the two groups.

### Experimental design

**Figure 1:**
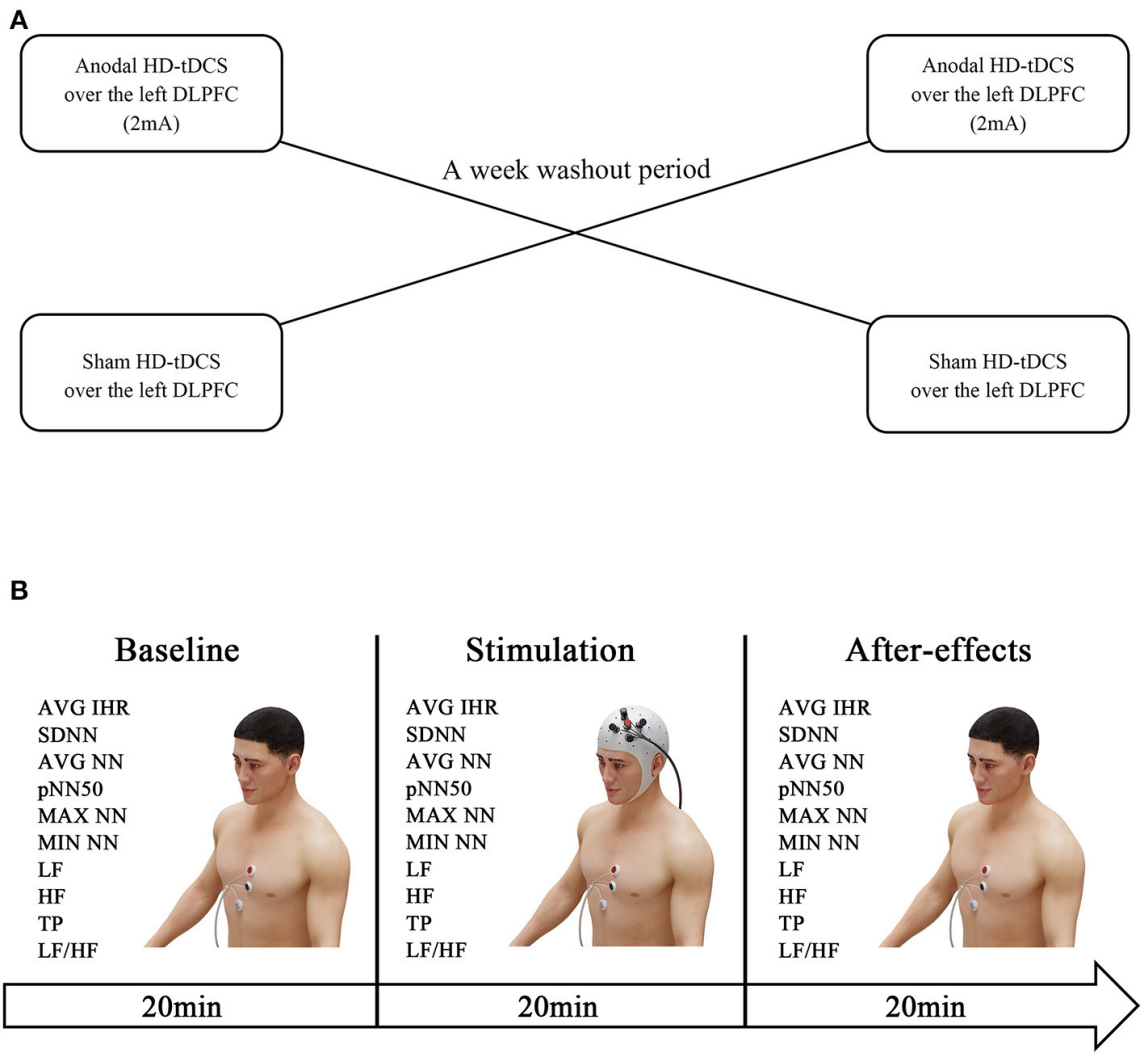
Experiment design. The HR and HRV data were measured at baseline (20 min before the HD-tDCS session). Subsequently, the participants were randomized to receive anodal HD-tDCS (2 mA) or sham HD-tDCS over the left DLPFC, and electrocardiogram recordings were obtained throughout the intervention. One week later, the interventions were swapped **(A)**. The after-effects were measured by evaluating the HR and HRV within 20 min post-treatment **(B)**.

This study adopted a randomized crossover design (Figure 1A). All participants were randomized to receive anodal HD-tDCS or sham HD-tDCS over the left DLPFC with a 1-week washout period. The participants were instructed to maintain their daily routine throughout the duration of the study. We continuously measured the HR and HRV data at baseline as time period t0 (20 min before a session of HD-tDCS), during the session as time period t1 (20 min), and 20 min after the session as time period t2 (Figure 1B).

The experiment was conducted in an isolated, quiet, well-lit room. During the course of the experiment, the participants were comfortably seated in a chair with arms and backrest in natural light. Constant temperature (23 °C) and relative humidity (40–60%) were maintained. This study was approved by The Ethics Committee of Nanjing Sports Institute (approval no. RT-2022-02) and was registered with the China Clinical Trial Registration Center (http://www.chictr.org.cn; no. ChiCTR2200061068). All experimental procedures were conducted in accordance with the guidelines for human medical research (Declaration of Helsinki).

### HR and HRV measurements

The electrocardiogram (ECG) signals were recorded by a wireless multichannel recording system (Plux Wireless Biosignals S.A., Lisboa, Portugal) at a sampling rate of 1000 Hz per channel with 16-bit precision. A three-electrode configuration was employed to record ECG. The red electrode was positioned at the third intercostal on the left of the sternum while the black electrode was positioned at the fourth intercostal or fifth intercostal (lower edge of the female breast) on the left of the sternum. The reference electrode was placed below the xiphoid process.

Before initiation of the experiment, the participants sat quietly for 5 min (resting phase). Then, ECG data were recorded during the three aforementioned periods throughout the experimental procedure. The recorded data were synchronized using measurement-equipment-matched software, which automatically determined the HR and HRV indexes from the recorded data and generated a report displaying the HR and HRV index data.

The average instant heart rate (AVG IHR) was adopted as an index of HR in statistical analysis. HRV indexes are usually categorized into time- and frequency-domain indexes (28). In the study, we measured the HRV indexes as follows. The time-domain indexes included the standard deviation of the normal-to-normal (NN) interval (SDNN), the average of the NN interval (AVG NN), the minimum NN interval (MIN NN), the maximum NN interval (MAX NN), and pNN50. The frequency-domain indexes included very low-frequency power (VLF, < 0.04 Hz), low-frequency power (LF, 0.04–0.15 Hz), high-frequency power (HF, 0.15–0.4 Hz), and the LF/HF ratio. The total power was the sum of the VLF, LF, and HF power. The data was recorded within three different 20-min time periods.

### Interventions

**Figure 2:**
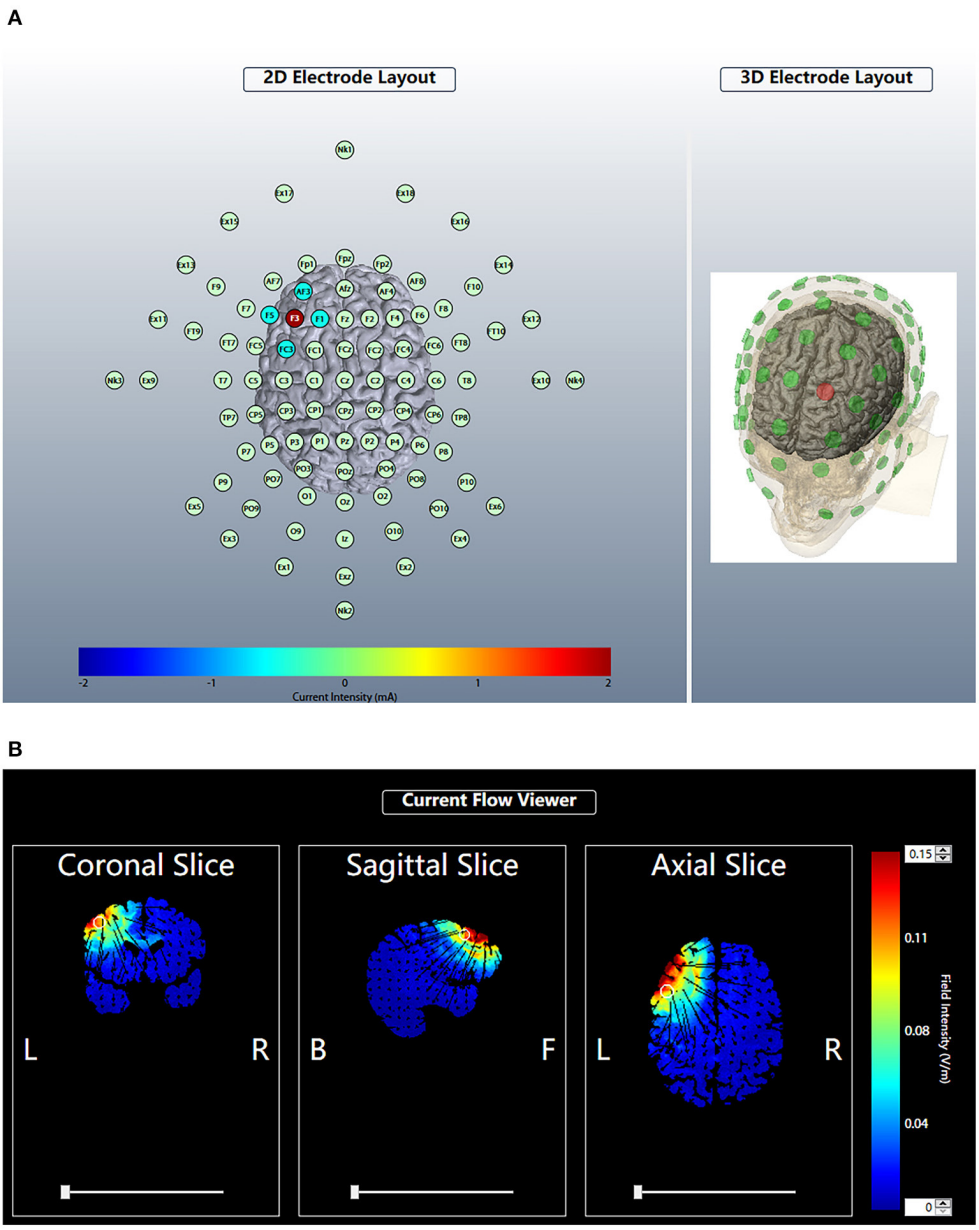
Brain modulation during HD-tDCS using the 4 × 1 ring configuration. According to the International 10-20 system, the anodal electrode was placed at F3. The four cathodal electrodes were placed on AF3, F5, FC3 and F1. The current intensity was 2 mA **(A)**. For each plane, we estimated the theoretical current flow in terms of the modeled electric field normal component (nE, V/m) at the cortex of the HD-tDCS electrode array by Soterix HD-Explore. Blue indicates zero electric field (0 V/m), while red indicates peak magnitude (0.15 V/m) **(B)**.

Anodal HD-tDCS: The device used a 4 × 1 HD-tDCS adaptor with five small circular electrodes (l cm^2^) (Soterix Medical, New York, NY, USA). The electrode montage we used in the study included the central anodal electrode placed on the left DLPFC (F3 position, International 10-20 system) and a total of four cathodal electrodes (AF3, F5, FC3, F1) separated from the central electrode by a distance of 3.5 cm placed around the central electrode to form a circular current loop after applying conductive paste. The left DLPFC is located 5 cm anterior to the primary motor cortex in a parasagittal plane (29). The stimulation was initiated after successfully completing the lead quality test. For each of the five electrodes, the lead quality values were verified to be < 1 quality unit, corresponding to actual impedance values less than 30 kΩ. The current intensity was set to 2 mA, and the time of stimulation was set to 20 min. For the sham stimulation, the intensity was slowly increased to 2 mA in the first 30 s, and then gradually lowered to 0 in 30 s; the other parameters were maintained consistent with the active stimulation. All participants were informed that they were receiving the active stimulation (Figure 2).

### Statistical analysis

The Jamovi (1.6.23.0) and Graphpad (8.4.0) software programs were used for statistical analysis. First, the ROUT (Q = 1%) function in GraphPad Prism was used to eliminate outliers and remove them from further research. Subsequently, a normal probability plot test was used to determine the normal distribution of the data. Descriptive statistics were used to summarize patient characteristics. When describing continuous variables, medians with interquartile ranges (IQR) were used for non-normally distributed data and means with standard deviations were used for data showing a normal distribution. To ascertain carryover effects, two-sample independent *t*-tests and the Mann-Whitney U test were first performed. Subsequently, we used a linear mixed effects model for the outcomes with a normal distribution (SDNN, AVG IHR, AVG NN) and a generalized linear mixed model for non-normally distributed data (pNN50, MAX NN, MIN NN, LF, HF, TP, LF/HF). We entered time (t0, t1, t2) and intervention (anodal HD-tDCS, sham HD-tDCS) as fixed effects and subject (intercept) effect as a random factor. All models tested the INTERVENTION × TIME interaction. Before analyzing the main and interaction effects, we performed an exploratory planned interaction contrast to investigate the effect of HD-tDCS on HR and HRV during the stimulation and recovery periods, using Bonforroni correction for pairwise comparisons. For significant main effects or interactions, *post-hoc* analyses were conducted and *p*-values were adjusted according to the Bonferroni correction method for multiple testing. The two-sided significance level was set at *p* < 0.05.

## Results

**Figure 3:**
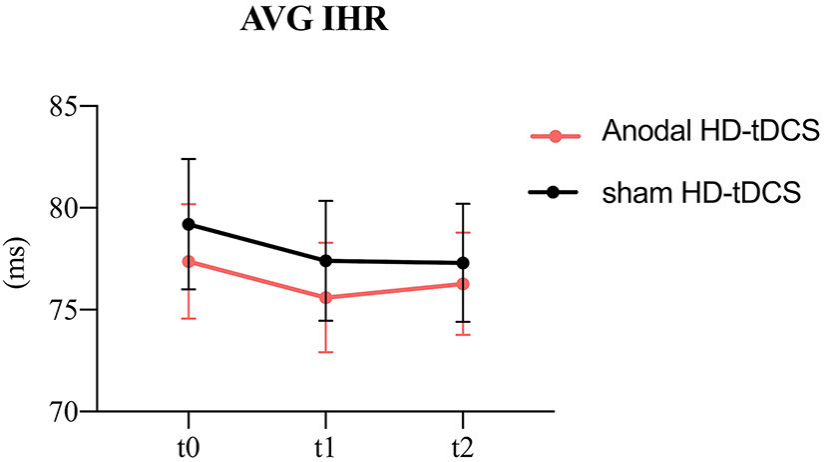
Evolution of heart rate. The figure shows the intervention × time interaction for HR. Effect of the intervention on AVG IHR. Graphs show the mean values with error bars indicating 95% confidence intervals. The red line represents the Anodal HD-tDCS group and the black line represents the sham stimulation group.

**Figure 4:**
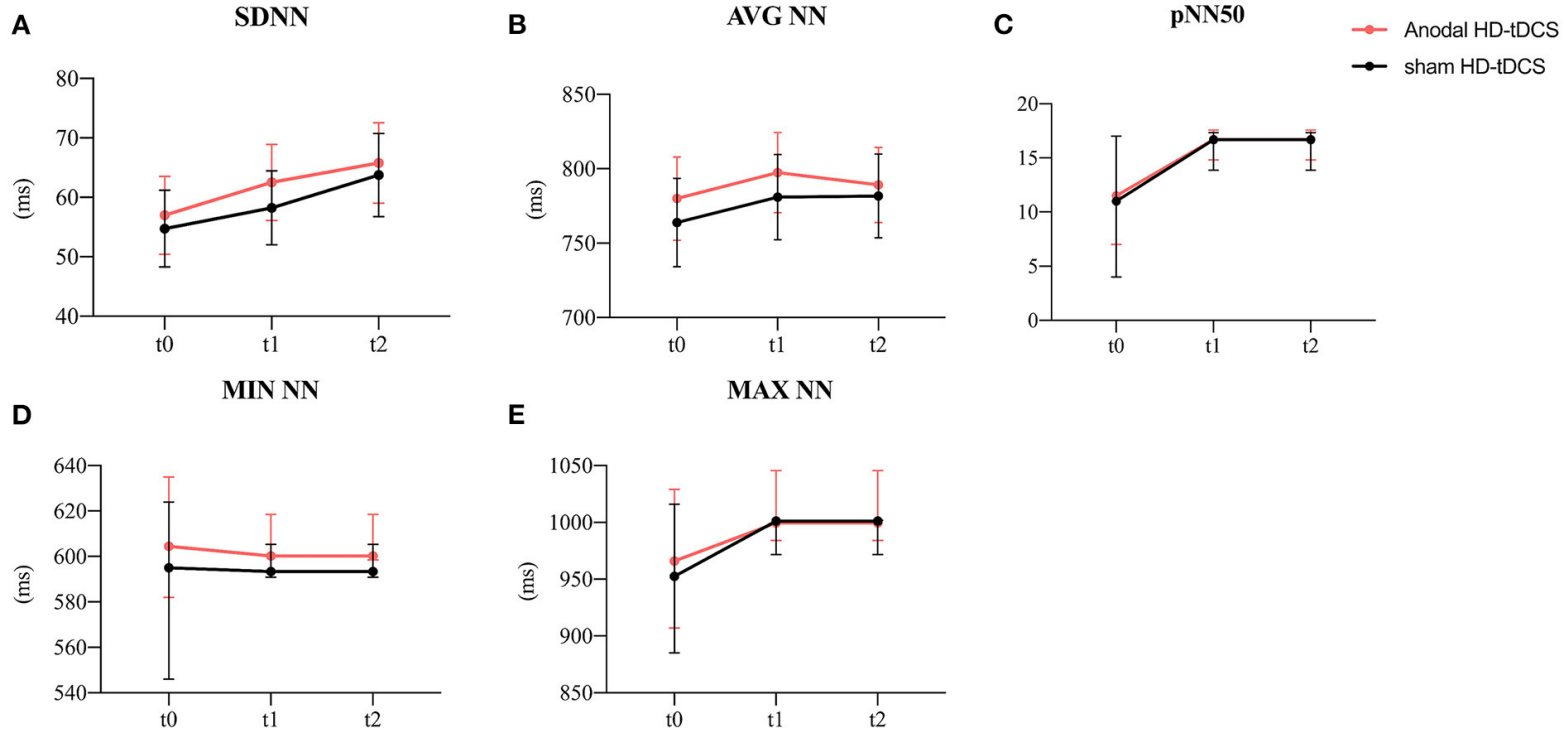
Evolution of time-domain heart rate variability. **(A)** Effect of the intervention on SDNN. Graphs show the mean values with error bars indicating 95% confidence intervals. **(B)** Effect of the intervention on AVG NN. Graphs show the mean values with error bars indicating 95% confidence intervals. **(C)** Effect of the intervention on pNN50. Graphs show the median values with error bars indicating 95% confidence intervals. **(D)** Effect of the intervention on MIN NN. Graphs show the median values with error bars indicating 95% confidence intervals. **(E)** Effect of the intervention on MAX NN. Graphs show the median values with error bars indicating 95% confidence intervals. The red line represents the Anodal HD-tDCS group and the black line represents the sham stimulation group.

**Figure 5:**
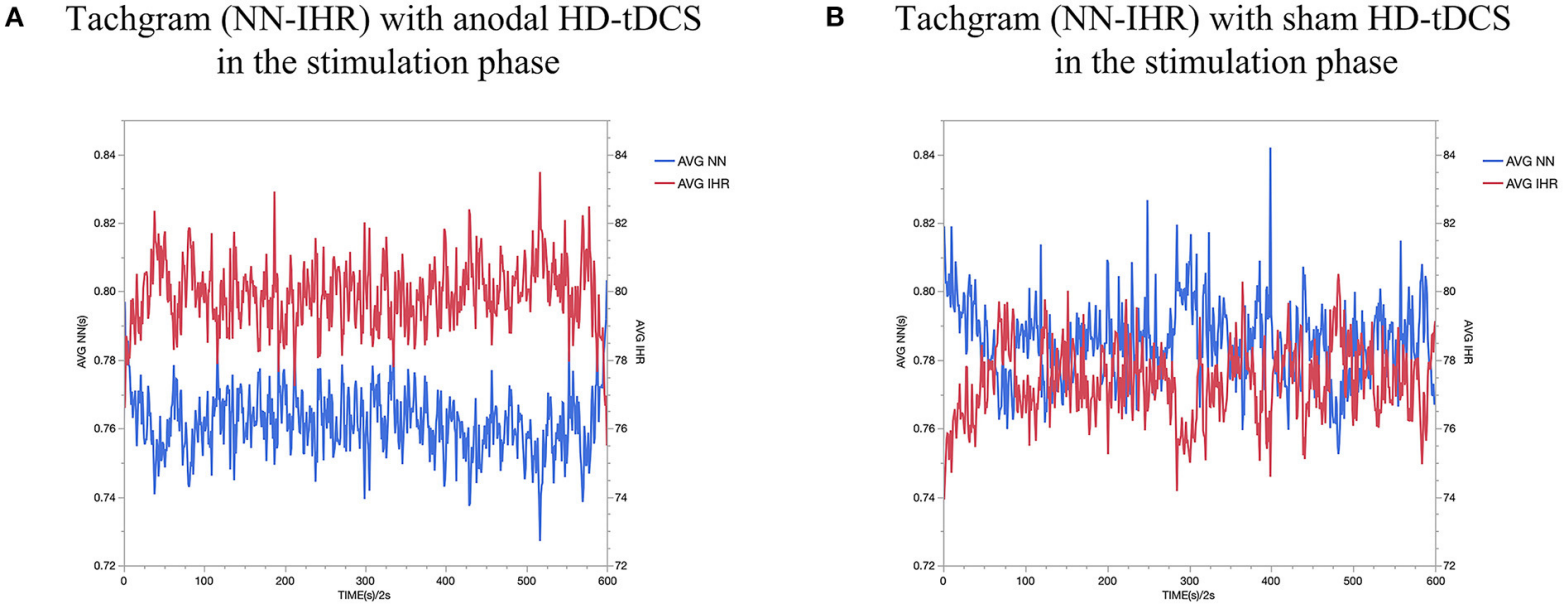
AVG NN interval and AVG IHR comparing Anodal HD-tDCS **(A)** vs sham HD-tDCS **(B)**. The red dash represents AVG IHR and the blue dash represents AVG NN.

**Figure 6:**
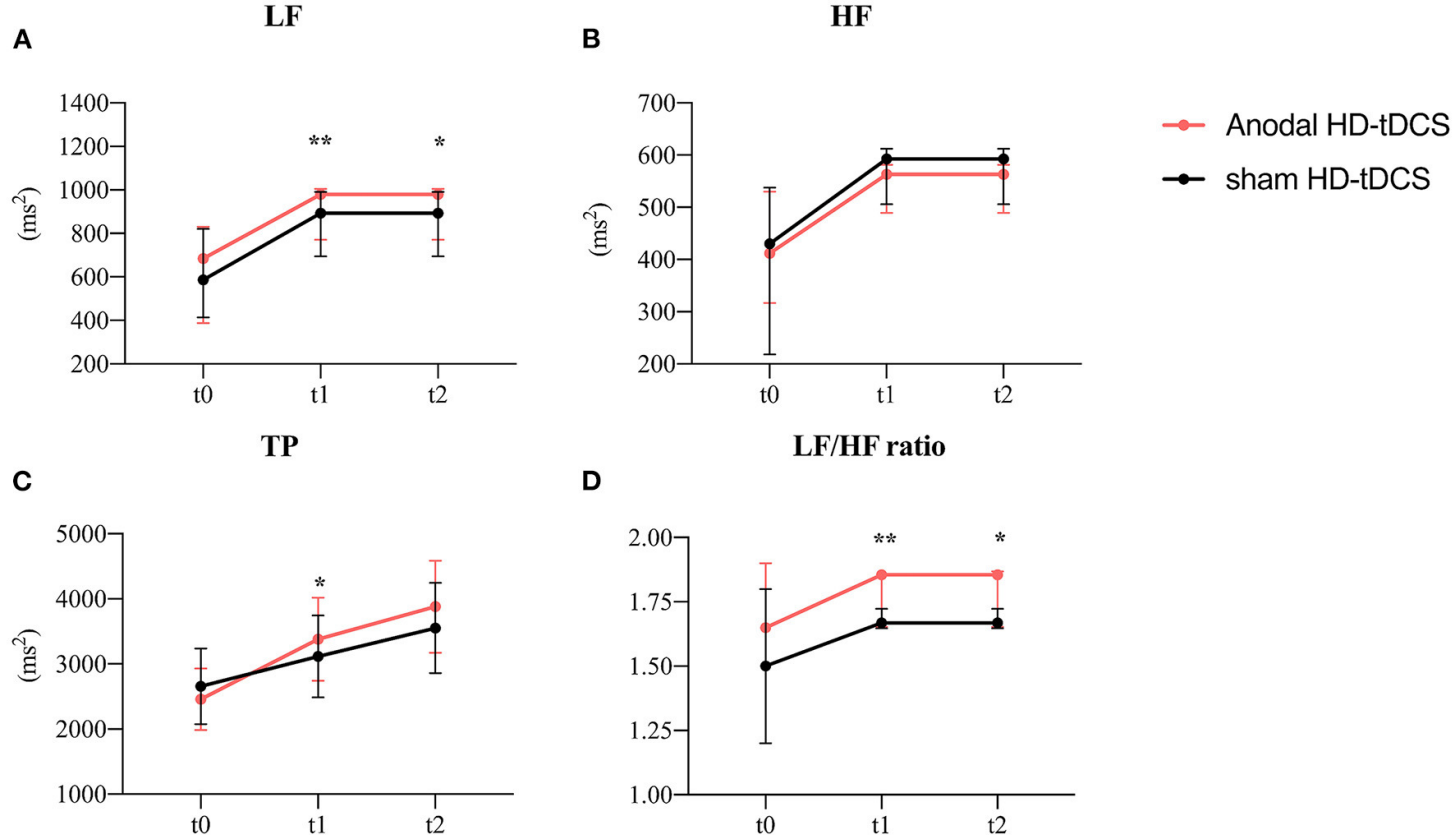
Evolution of frequency-domain heart rate variability. **(A)** Effect of the intervention on LF. Graphs show the median values with error bars indicating the 95% confidence intervals. **(B)** Effect of the intervention on HF. Graphs show the median values with error bars indicating the 95% confidence intervals. **(C)** Effect of the intervention on TP. Graphs show the median values with error bars indicating the 95% confidence intervals. **(D)** Effect of the intervention on LF/HF. Graphs show the median values with error bars indicating the 95% confidence intervals. Asterisk indicates that the intervention differed at the between-group level at this time point (*p* < 0.05), double asterisks at 0.01 level. The red line represents the Anodal HD-tDCS group and the black line represents the sham stimulation group.

After excluding all outliers that did not alter the significance, Table 1 presents the demographics and the characteristics of HRV at t0. After a week of washout, two-sample *t*-tests and a Mann-Whitney U test showed no significant difference between the baseline values of the two sessions. No participants reported any adverse effects. The trends of HRV in the two interventions are reported in Figures 3–6.

**Table 1:** Baseline characteristics of the subjects.

**Characteristic**		***N***
Age, mean (SD), yr	22(1.3)	40
Sex, male:female	16:24^a^	40
Height, mean (SD), cm	167.2(8.5)	40
Weight, mean (SD), kg	61.1(15.1)	40
BMI, mean (SD)	21.7(3.9)	40
AVG IHR, mean (SD)	78.3 (9.4)	80
**Heart-rate variability**		
SDNN, mean (SD), ms	55.9 (20.1)	79
AVG NN, mean (SD), ms	772 (90)	80
pNN50, median (IQR), %	11 (3, 20)	78
MAX NN, median (IQR), ms	959 (878.5, 1036)	79
MIN NN, median (IQR), ms	599 (545, 645.5)	79
LF, median (IQR), ms^2^	605 (346.8, 966.5)	76
HF, median (IQR), ms^2^	415 (217, 635)	75
TP, median (IQR), ms^2^	2229 (1245, 3356)	77
LF/HF ratio, median (IQR)	1.5 (1.0, 2.1)	76

a The number of subjects for the respective gender.

N, Number of samples included in the statistical analysis.

### HR measurements in different interventions

The model of the AVG IHR (Figure 3) revealed the intervention main effect (F = 7.04, *p* = 0.009) and the time main effect (F = 3.60, *p* = 0.029). The anodal HD-tDCS induced a decrease in AVG IHR in comparison with the sham HD-tDCS intervention.

In terms of time factor, The AVG IHR was significantly decreasing at t1compared to t0 (*p* = 0.040).

### HRV time-domain measurements in different interventions

The model analysis of data revealed that only the time main effect was significant for SDNN and MAX NN (Figure 4A, SDNN: F = 10.29, *p* < 0.001; Figure 4E, MAX NN: χ^2^ = 3.60, *p* = 0.029). The SDNN was significantly increasing at t2 (*p* < 0.001) while the MAX NN was significantly increasing at t1 (*p* < 0.001) compared to t0. The model of the AVG NN revealed the intervention main effect (Figure 4B, F = 5.44, *p* = 0.020) and the time main effect (F = 3.36, *p* = 0.037). The anodal HD-tDCS induced an increase in AVG NN in comparison with the sham HD-tDCS intervention. In terms of time factor, The AVG NN was significantly increasing at t1compared to t0 (*P* = 0.044). No main or interaction effect was found for pNN50 and MIN NN (Figures 4C,D). The detailed comparison results are in Table 2.

**Table 2:** Comparison of the duration and frequency of heart-rate variability with different interventions and at different points.

	**Between–intervention differences at different points. estimate (95% CI)**					
**Group**	**T0**	***p-*value**	**T1**	***p*-value**	**T2**	***p*-value**
	**tDCS vs. sham**		**tDCS vs. sham**		**tDCS vs. sham**	
**Outcomes for HR**						
AVG IHR	−1.83 (−3.82,0.17)	0.073	−1.80 (−3.80,0.20)	0.077	−1.03 (−3.02,0.97)	0.312
**Outcomes for the time domain of HRV**						
SDNN (ms)	2.17 (−3.32,7.66)	0.437	4.21 (−1.28,9.70)	0.132	1.99 (−3.50,7.48)	0.476
AVG NN (ms)	16.13 (−3.38,35.63)	0.105	16.48 (−3.03,35.98)	0.097	7.35 (−12.16,26.86)	0.458
pNN50 (%)	−0.07 (−0.28, 0.14)	0.520	−0.06 (−0.26, 0.15)	0.573	−0.03 (−0.24, 0.18)	0.778
MAX NN (ms)	19.97 (−14.11,54.06)	0.251	32.75 (−1.73,67.22)	0.063	1.95 (−37.66,33.76)	0.915
MIN NN (ms)	12.48 (−4.77,29.74)	0.156	12.73 (−5.40,30.87)	0.169	4.57 (−13.75,22.89)	0.625
**Outcomes for the frequency domain of HRV**						
LF (ms^2^)	11.76 (−45.96,69.47)	0.689	91.26 (23.50,159.02)	**0.008**	80.00 (9.55,150.45)	**0.026**
HF (ms^2^)	0.25 (−1.08,1.58)	0.710	0.06 (−1.44,1.32)	0.928	0.52 (−1.91,0.87)	0.461
TP (ms^2^)	1.10 (−1.92,4.11)	0.476	3.53 (0.24,6.82)	**0.035**	2.84 (−0.67,6.35)	0.112
LF/HF ratio (%)	0.07 (−0.09,0.22)	0.399	0.21 (0.07,0.34)	**0.004**	0.26 (0.06,0.45)	**0.012**

Bold text, *P* < 0.05. Bonforroni correction was used for pairwise comparisons.

As shown in Figure 5 for all the collected subjects. In this figure, the red dash represents AVG IHR and the blue dash represents AVG NN. As a result of stimulating the left DLPFC, we observed a decrease in HR and an increase in AVG NN during stimulation.

### HRV frequency-domain measurements in different interventions

A plan interactive contrast showed that the LF, LF/HF, and TP were higher at t1 than in the sham group. In addition, the LF and LF/HF ratio were still higher at t2 than in the sham group. The difference is statistically significant after the Bonforroni correction. (Detailed results are presented in Table 2). A generalized linear mixed model analysis revealed that no fixed effect was significant for HF (Figure 6B, *p* > 0.05). LF, LF/HF ratio, and TP were higher in the tDCS stimulated group than the sham stimulated group, and the difference between the groups was statistically significant (Figure 6A, LF: intervention [χ^2^ = 9.91, *p* = 0.002], time [χ^2^ = 19.65, *p* < 0.001]; Figure 6D, LF/HF ratio: intervention [χ^2^ = 13.16, *p* < 0.001); Figure 6C, TP: intervention [χ^2^ = 6.37, *p* = 0.012], time [χ^2^ = 24.32, *p* < 0.001]). A *post-hoc* test showed that the LF, LF/HF, and TP were higher at t1 than in the sham group. In addition, the LF and LF/HF ratio were still higher at t2 than in the sham group (detailed results are presented in Table 2).

**Figure 7:**
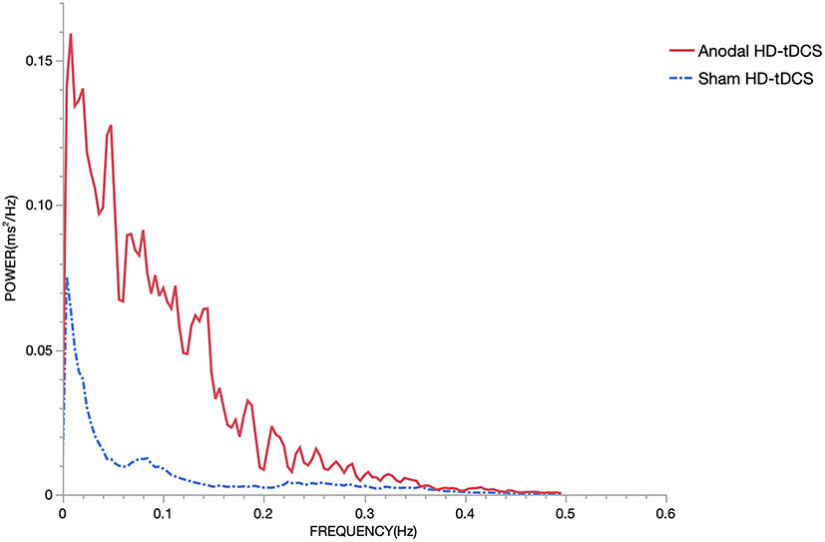
Power spectrum. The sampling rate of the ECG was 1000 Hz. There is an increment of the power during the stimulation using anodal HD-tDCS compared to sham HD-tDCS. The red solid line represents the anodal HD-tDCS group and the blue dashed line represents the sham stimulation group.

As shown in Figure 7, The mean results collected from all subjects showed some differences in HRV as determined from spectral analysis in the TP. HD-tDCS, particularly after stimulation of the left DLPFC, induced a slight increase in the TP compared to the sham condition. The quantitative changes in the power spectrum of the HRV proved that the cardiovascular control mechanism was altered during HD-tDCS.

## Discussion

Existing research has mainly focused on the changes in executive ability, cognitive ability, or interest- and emotion-related performance as a result of tDCS stimulation in the corresponding brain regions (30–32). Even in studies reporting HR and HRV data, these parameters were not the primary efficacy measures (33). At present, the effects of NIBS intervention on HR and HRV are not clear. This study aimed to explore the HR and HRV changes anodal HD-tDCS over the DLPFC. We utilized the HD-tDCS technology, which uses a concentric ring electrode configuration with the central electrode placed at the stimulation target and four return electrodes around, and the new electrode arrangement proved to be effective in inducing cortical plasticity (23, 34). The central anodal electrode causes membrane depolarization and activates Na^+^ and Ca^2+^ voltage-gated channels, which may result in LTP-like plasticity by increasing cortical excitability. It has a more focused electric field and high spatial resolution to fulfill the needs of precision stimulation. The electrode placement generates a peak in the focused electric field under the central electrode, thus reducing the spread of current into the brain (35). The major finding of this study is that in healthy young people, anodal HD-tDCS over the left DLPFC could decrease HR and modulate HRV. Left DLPFC appears to be a target with potential applications and research value, and these findings can be used to modulate HR and HRV in non-invasive brain neuromodulation (27, 36).

In this study, in terms of the overall trend of HR, anodal HD-tDCS caused a decrease in AVG IHR by increasing cortical excitability over the left DLPFC. It has been shown that the medulla oblongata and spinal cord nuclei within the brain stem are involved in the control of cardiovascular responses. Brain stem lesions could result in impaired cardiac function and autonomic dysreflexia (37). In addition to this, the PFC is referred to as the visceral motor cortex, and it regulates cardiovascular response by transmitting information through bidirectional connections to the brain stem (38, 39). Thus, the activation of PFC has been shown to influence cardiac outflows and respiratory function. The increased excitability of the PFC in humans enhances limbic connectivity and dominates the limbic system through frontal tonic inhibition to decrease the HR (40). Some studies have demonstrated that the PFC inhibits overactivity in limbic structures, which overexcitation can cause the autonomic imbalance and increase in HR (41, 42). In addition, pharmacological blockade of the PFC would result in an increase in HR (43). Meanwhile, AVG NN is inversely proportional to the IHR. The increase in AVG NN coincided with the reduction in IHR (44). Similar results were obtained in our study.

For the frequency domain of the HRV, the LF/HF ratio can reflect the sympathovagal balance (45). We found that anodal HD-tDCS over the left DLPFC induces a significant increase in LF/HF in comparison with the sham stimulation, indicating that the sympathetic input of the ANS is higher than the parasympathetic input in normal conditions. Excitatory high-frequency rTMS over the left DLPFC attenuates the HPA axis response to reduce overall cortisol secretion, which was reflected in higher HRV (7). The effect can be explained by the mechanism of brain-heart communication (4). The neurovisceral integration (NVI) model proposes a significant association between cardiac responses and cortical structures (46), such as the medial pre-frontal cortex (MPFC), the insular cortex, and higher subcortical regions. These interconnected areas are called the central autonomic network (CAN), which is responsible for regulating cardiovascular function in the ANS and HPA axis (47, 48). The PFC, as an important brain region in CAN, participates in inhibitory control to regulate the cardiac efferent pathways in emotion processing and self-regulation (49, 50). The sympathoexcitatory circuit in the amygdala is inhibited by increased excitability of PFC, which reduces parasympathetic depression and ultimately leads to a lower HR (51).

Previous studies have suggested that different NIBS stimulation targets have different intervention effects on the regulation of HR and HRV (27, 52, 53). Carnevali et al. discovered that decreased HR at rest may be caused by anodal tDCS (2 mA) applied across the left DLPFC in healthy adult males to regulate autonomic function (54). Brunoni et al. performed anodal tDCS (1.5 mA) over the left DLPFC in healthy participants and found that a significant increase in HF and vagal activity was reflected in the HRV indices (25). Our results seem to be in agreement with the above studies. However, several studies yielded results that are not similar to ours. A study on anodal tDCS (2 mA) treatment over the left or right temporal cortex showed no significant differences in HR about the effects of the cardiovascular response (55). Vandermeeren and colleagues proposed that anodal tDCS (2 mA) applied over the midline frontal cortex in 30 healthy volunteers resulted in a slight decrease in respiratory rate and a steady increase in blood pressure, but the HR remained stable during the session. There were no significant differences between the anodal and sham tDCS groups (56). These different conclusions might be related to the different stimulation targets.

Our findings may have several potential clinical applications. Patients with chronic stress or post-traumatic stress disorder (PTSD) under long-term stress have a higher HR and blood pressure, resulting in an increased risk of cardiovascular disease (8, 57). Anodal HD-tDCS over the left DLPFC could presumably be used as a potential adjuvant therapy for chronic stress or PTSD. Additionally, for the majority of chronic heart failure patients, the presence of long-term tachycardia can induce myocardial ischaemia, aggravate symptoms, and affect quality of life (58). Perhaps this is a strategy that can be utilized to decrease rapid HR and delay disease progression in patients with heart failure. As is well known, HR is recognized as an important index to assess exercise-induced fatigue. When resting HR is too high, a higher maximal rate of HR increase during exercise is associated with decreased performance due to acute fatigue (59). Angius et al. performed anodal tDCS (2 mA) over the left DLPFC to observe the changes in inhibitory control and endurance performance of participants during cycling exercise performance and found significant reductions in HR and ratings of perceived exertion (27). As a result, anodal HD-tDCS could perhaps be used to enhance training intensity and endurance by reducing HR in athletes.

This preliminary study provides a basis for investigating the influence of HD-tDCS over the left DLPFC on cardiac response in the future. Additionally, the unclear aspects related to brain-heart communication in people with different ages and different diseases need to be verified in further relevant randomized clinical trials. Since 24 h ECG monitoring was not performed, there was a lack of long-term (≥24 h) HRV measurements. To clarify how cortical activity modulates cardiovascular function, further research in imaging, physiology, and biochemistry is anticipated.

## Conclusion

Anodal HD-tDCS on the left DLPFC affects cardiovascular and ANS activity. It causes a reduction in the HR and an increase in the NN interval and LF/HF ratio, and modulates the sympathovagal balance. Based on brain-heart communication, HD-tDCS is proposed as a potential intervention strategy to affect cardiovascular response and autonomic function. This lays the foundation for further investigations and provides a clinical basis to explore the application of HD-tDCS in autonomic disorders, cardiovascular diseases or athletes.

## Data availability statement

The raw data supporting the conclusions of this article will be made available by the authors, without undue reservation.

## Ethics statement

The studies involving human participants were reviewed and approved by the Ethics Committee of Nanjing Sports Institute. The patients/participants provided their written informed consent to participate in this study.

## Author contributions

YS and ZG conceived and designed the study. ZG, WC, JD, and YG performed the study and collect materials. QL, KX, and BX analyzed the results. WD, ZG, QL, and SH wrote the manuscript. YS, WD, JD, and YZ helped coordinate the study and reviewed the manuscript. All authors contributed to the article and approved the submitted version.

## Funding

This study was funded by the National Key Research & Development Program of China (2022YFC2009700, 2022YFC2009701, and 2022YFC2009702).

## Conflict of interest

The authors declare that the research was conducted in the absence of any commercial or financial relationships that could be construed as a potential conflict of interest.

## Publisher's note

All claims expressed in this article are solely those of the authors and do not necessarily represent those of their affiliated organizations, or those of the publisher, the editors and the reviewers. Any product that may be evaluated in this article, or claim that may be made by its manufacturer, is not guaranteed or endorsed by the publisher.
